# Private well stewardship within a rural, agricultural Latino community: a qualitative study

**DOI:** 10.1186/s12889-020-08963-4

**Published:** 2020-06-05

**Authors:** Kori VanDerGeest, Linda K. Ko, Catherine Karr, Elizabeth Torres, Dennise Drury, Elena Austin

**Affiliations:** 1 Department of Environmental and Occupational Health Sciences, University of Washington School of Public Health, Seattle, WA USA; 2 Division of Public Health Sciences, Fred Hutchinson Cancer Research Center, Seattle, WA USA; 3 Department of Health Services, University of Washington School of Public Health, Seattle, WA USA; 4 Pacific Northwest Agricultural Safety and Health Center, University of Washington School of Public Health, Seattle, WA USA; 5 Department of Pediatrics, University of Washington School of Medicine, Seattle, WA USA; 6 Northwest Communities Education Center/Radio KDNA, Granger, WA USA

**Keywords:** Private wells, Latinos, Household water treatment, Nitrate, Disparities, Agriculture, Drinking water

## Abstract

**Background:**

Nitrate contamination in groundwater disproportionately impacts agricultural Latino communities, creating a significant hazard for Latinos that rely on private wells. Private well users must conduct water testing and other well stewardship behaviors to ensure that their well water is safe to drink. This study sought to identify the key factors impacting private well water testing behavior in rural, agricultural Latino communities.

**Methods:**

We conducted 4 focus groups with private well users, 2 in Spanish and 2 in English. We recruited 37 participants from the Lower Yakima Valley, Washington State, a rural, agricultural community with a large Latino population and elevated nitrate concentrations in groundwater. A semi-structured interview guide was developed to capture factors impacting testing as guided by the Risk, Attitudes, Norms, Ability, and Self-Regulation (RANAS) model. Inductive thematic analysis was conducted by two coders to identify common themes.

**Results:**

Themes emerged around the factors impacting well stewardship, including well water testing, treatment, and maintenance, and were not specific to nitrate contamination. Private well users reported many of the same factors reported in other communities, with the exception of home repair experience and challenges around landlords and neighbors on shared wells, which have not been reported previously. In addition to landlords and neighbors, lack of actionable information, economic limitations, and lack of technical support emerged as factors that made well stewardship burdensome for individuals. The majority of participants reported using bottled water, including many who used point-of-use or point-of-entry water treatment systems.

**Conclusions:**

The burden of well stewardship in rural, agricultural Latino communities may suggest the need for interventions at the community, county, or state levels and not at the individual level alone. Additionally, the role of landlords, neighbors on shared wells, and home repair experience in well stewardship represent important areas of exploration for researchers and public health practitioners.

## Background

The US Safe Drinking Water Act does not regulate private wells, leaving over 42 million US residents with little regulatory oversight of their water quality [1]. Private well users are responsible for ensuring that their water is safe to drink. According to a survey conducted by the US Geological Survey (USGS) in 48 states, 23% of domestic wells in the US contained one or more contaminants at a concentration exceeding a human health standard [2]. Nitrate is the most common anthropogenic contaminant in private wells [2], originating from synthetic fertilizers, fossil fuel combustion, animal waste, and wastewater [3]. The maximum contaminant level for nitrate in regulated drinking water sources is 10 mg/L NO_3_-N [4]. Consuming water above this concentration may lead to developmental effects, gastrointestinal cancer, and methemoglobinemia, which can be fatal in infants if not treated [5].

Nitrate contamination in drinking water supplies disproportionately impacts Latino communities [6, 7]. Public water systems in the top quartile of percent Latino residents served are nearly three times more likely to exceed 5 mg/L of nitrate than public water systems in the bottom quartile [7]. These disparities may occur because agriculture is the largest input of nitrogen in US water resources [3], and 83% of farm workers are Latino [8]. Public water systems with groundwater sources have higher nitrate concentrations than those with surface water sources [7], suggesting that Latino communities relying on private wells may also be disproportionately impacted by nitrate contamination. The Lower Yakima Valley (LYV) in Central Washington is one such community. LYV is home to a rural, agricultural Latino community where approximately 34% of residents rely on private well water [9]. A 2017 USGS survey found that 26% of private wells in LYV had a nitrate concentration that exceeded the maximum contaminant level at least once over a nine-month period [10]. Given the extensive well water contamination in this community, it is critical to understand how private well users ensure that their water is safe to drink.

Private well users must conduct a number of activities to keep their water safe, including proper construction, well maintenance, periodic well water testing, and water treatment as necessary. Altogether, these behaviors are referred to as well stewardship. Literature on factors impacting well stewardship behavior is extensive [11–18]. Research shows that private well users are often satisfied with the quality of their well water and confident that it is safe to drink [11–13]. Many note its good taste, smell, and clarity [12, 13] and may not see the need for testing and treatment due to low perceived risk of water contamination and related health effects [12]. Other factors that hinder testing and treatment include cost, inconvenience, not knowing how to test, and lack of social norms [11, 12, 14–16]. The majority of these studies were conducted in regions with primarily non-Latino White populations and there is little literature on the factors impacting well stewardship behavior in Latino populations.

A key component of well stewardship is well water testing, but some private well users do not test their wells [17] and few test at recommended frequencies [14, 18]. The Centers for Disease Control and Prevention (CDC) recommends that well water users test for nitrate every year [19]. In one of the few studies on well water testing in majority Latino communities, 52% of survey respondents in agricultural Central Washington reported never testing their wells despite the prevalence of high nitrate concentrations in the study area [20]. Although testing rates may be low in rural, agricultural Latino communities, there is inadequate research examining the factors impacting well water testing within this population. Guided by the Risk, Attitudes, Norms, Ability, and Self-Regulation (RANAS) model [21], this study examined the key factors impacting private well water testing behavior among well water users residing in predominantly Latino communities in rural, agricultural areas.

## Methods

We conducted 4 focus groups between November 2018 and January 2019 with private well users in LYV, WA: 2 in Spanish and 2 in English. We used purposive sampling to recruit participants, sampling for both Spanish and English speakers based on self-reported language preference for focus group discussion. Individuals were eligible for participation if they reported using a private well, were 18 years or older, and had one of the following individuals in their household: a child, a pregnant individual, or an adult 65 years or older as they are highly susceptible to health effects related to nitrate and total coliform, which are common well water contaminants in LYV. We included non-Latinos to capture perceptions of well stewardship in the broader community, recognizing that many Latinos in rural, agricultural areas do not live in racially/ethnically homogenous communities. We excluded individuals who had attended a meeting of the local groundwater management committee as their perceptions may differ from the general LYV population. Individuals received $25 for participating in the focus groups. The University of Washington Human Subjects Division determined that this study was exempt from federal human subject regulations.

### Setting

LYV is a rural, agricultural region where residents live in small cities and unincorporated communities across the valley. As reported by the 2018 American Community Survey, the region has a total population of 61,356, of which 72.0% is Latino and 25.8% is non-Latino White [22]. LYV is home to a large immigrant community: 25.2% of the population is foreign born [23], of which 94.4% were born in Mexico [24].

### Recruitment

We used multiple methods to recruit participants including radio, flyers, door-to-door canvassing, community meetings and participant referrals in partnership with community-based organizations. We supplemented with cold-calls, calling individuals who had participated in a free well water testing program organized by the county. Two research team members (KV and ET) recruited the focus group participants. ET, an English/Spanish bilingual and bicultural research team member, recruited all Spanish-speaking participants. Before the focus groups, participants completed a ten-minute phone questionnaire on demographics and well stewardship behavior. Verbal consent was obtained from all participants at the start of the focus group sessions.

### Focus group guide

Focus group questions were informed by the Risk, Attitudes, Norms, Ability and Self-Regulation (RANAS) model for water-related health behaviors [21], which has been applied in well stewardship studies previously [14, 15, 25]. The model is composed of five factor blocks, which are listed in the model name. Each block contains psychosocial factors, such as self-efficacy in the ability factor block, which are drawn from health behavior theories such as the Health Belief Model, Integrated Behavioral Model, and Social Cognitive Theory. In the first segment of the moderator guide, questions covered participants’ perceptions of well water quality, water-related health risks, and well water testing. In the second segment, participants read about well water testing from a fact sheet developed by a health agency (see Additional File 1). The portions of the fact sheet read with participants included the agency’s recommendations for nitrate and coliform bacteria testing; cost, procedures, and recommended frequencies for testing; and methemoglobinemia resulting from the consumption of water with elevated nitrate concentrations. After reading the fact sheet, focus group questions focused on factors impacting well water testing behavior and intentions to test. All focus group questions were reviewed for cultural appropriateness by the research team, of which three team members were bicultural and bilingual in English and Spanish and one person was a member of the community.

### Data collection

Focus groups lasted 1.5 to 2 h and were convened at a local community center. ET moderated all four sessions and has prior experience moderating focus groups. Focus group discussions were audio recorded, transcribed verbatim, and checked for accuracy. Translation from Spanish to English was performed by a certified translator.

### Analysis

The transcripts were analyzed using inductive thematic analysis and ATLAS.ti Version 8 software. KV developed an initial codebook by identifying main ideas in the transcripts, clustering similar ideas into groups, and naming and defining the groups. All other members of the research team reviewed the codebook and provided feedback. KV and DD used the final codebook to independently code the transcripts and met regularly to reconcile discrepancies. ET, a member of the community, attended reconciliation meetings to provide contextual information and resolve coding disagreements. Following the methods outlined by Braun and Clark [26], we used thematic mapping to identify themes across codes. We constructed an initial map illustrating the relationships between codes. Then we revisited the transcripts, revising the maps until the concepts formed clear and succinct themes that accurately represented the data. Each theme represented a factor impacting behavior, which we defined as a concept that appeared to support or hinder behavior or intentions to act, as expressed by study participants.

## Results

Thirty-seven individuals (Table 1) participated in 4 focus groups, with 20 Spanish-speaking participants and 17 English-speaking participants. Each focus group had 7 to 11 participants. All the Spanish-speaking participants and a majority of English-speaking participants (11/17) were Latino. The remaining participants were non-Latino White. Several participants were renters (3/35) and 7 out of 35 participants shared their well with at least one other household. Over half of the participants (20/35) had ever tested their well water. Many treated their water with either a point-of-use (POU) or point-of-entry (POE) system (23/35), though only 2 out of 35 participants used a water treatment system capable of removing nitrate, such as a reverse osmosis system. Many participants purchased bottled water (24/35), including 12 individuals who also reported using a POU or POE system.

**Table 1:** Participant demographics and well stewardship characteristics (*n* = 37)

Characteristic	Total n (%)	Spanish language groups n (%)	English language groups n (%)
Number of participants	37 (100)	20 (54)	17 (46)
Gender			
Female	19 (54)	9 (50)	10 (59)
Male	16 (46)	9 (50)	7 (41)
Age (in years), mean (SD)	49 (14)^a^	43 (10)^a^	54 (16)^a^
Race			
Latino	29 (83)	18 (100)	11 (65)
Non-Latino White	6 (17)	0 (0)	6 (35)
Household Income			
< $25,000	11 (31)	9 (50)	2 (12)
$25,000 to $50,000	13 (37)	8 (44)	5 (29)
> $50,000	10 (29)	0 (0)	10 (59)
Declined to answer	1 (3)	1 (6)	0 (0)
Education			
Grade school or junior high	11 (31)	10 (56)	1 (6)
High school or GED	11 (31)	6 (33)	5 (29)
Trade school, associate’s degree, or college	10 (29)	1 (6)	9 (53)
Graduate school	3 (9)	1 (6)	2 (12)
Employment status			
Full time	16 (46)	7 (39)	9 (53)
Part time	10 (29)	5 (28)	5 (29)
Not employed	9 (26)	6 (33)	3 (18)
Home ownership			
Owner	32 (91)	15 (83)	17 (100)
Renter	3 (9)	3 (17)	0 (0)
Number of houses on well			
1	28 (80)	17 (94)	11 (65)
2	6 (17)	0 (0)	6 (35)
3–14	1 (3)	1 (6)	0 (0)
Have ever tested well water^b^	20 (57)	8 (44)	12 (70)
Mitigation used			
Bottled water	24 (69)	14 (78)	10 (59)
POU or POE treatment	23 (66)	12 (67)	11 (65)
Boil	3 (9)	1 (6)	2 (12)
None	2 (6)	0 (0)	2 (12)
POU or POE treatment			
Carbon filtration	13 (37)	10 (56)	3 (18)
Water softener	6 (17)	2 (11)	4 (24)
Particle filter	5 (14)	0 (0)	5 (29)
Reverse osmosis	2 (6)	1 (6)	1 (6)
Unknown	2 (6)	2 (11)	0 (0)

*SD* standard deviation, *POU* point-of-use, *POE* point-of-entry

^a^Mean and standard deviation

^b^The majority of English-speaking participants (59%) were recruited from a list of participants in a county private well water testing program

Demographic and well stewardship characteristics data missing for 2 participants

Participants across all four focus groups had lively discussions about well stewardship. Eight themes emerged around the factors impacting well maintenance, treatment, and bottled water use in addition to testing, the study’s original focus. These factors included concerns about water contamination, knowledge of agricultural sources, home repair experience, lack of actionable information, desire to protect family, economic limitations, lack of technical support, and landlords and neighbors. Themes differed little between the Spanish and English language groups with the exception of home repair experience, which was present only in the Spanish language groups.

### Concerns about water contamination

Participants had extended and dynamic discussions about well water contamination in which many spoke with a sense of concern, worry, or suspicion. Many stated that they did not drink their well water, but did use it for cooking, cleaning, or gardening. Participants across all focus groups expressed concern about contamination from nearby agricultural activities or water that looked, tasted, or smell bad. Participants asked many questions and one male Spanish-speaking participant explained, “I think we are here for the same purpose, because I also have no certainty that the water from the well is good [...] and for that reason we’re here because we want to know.” Several participants reported that their water looked and tasted good. Despite positive perceptions of their well water, these participants described water contamination as a concern that “sits in the back of your mind” (from an English focus group) and emphasized the need to be aware of well contamination issues.

### Knowledge of agricultural sources

Conversations between participants, particularly in the English language groups, demonstrated a knowledge of agricultural practices as a major source of well water contamination in the area. English-speaking participants described the processes that transport nitrate through the environment, discussing manure lagoons at industrial dairies, the use of manure to fertilize crop fields, infiltration into groundwater, and the impact of well depth and water table height on water quality. When discussing potential sickness from well water, one English speaker relied on her knowledge of the sources and transport of contamination.

*“Our water doesn’t taste horrible if we bypass the filter. However, we know that our water table is really high and we’re virtually surrounded by dairies. And so it crosses your mind. You think how much of what’s being sprayed right next door is infiltrating the ground around us and then seeping into the water table.”*

Both English and Spanish speakers expressed concern about contamination from pesticide application on crop fields and stated that well water should be tested for pesticides and other agricultural chemicals. Spanish-speaking participants shared concerns about pesticides from nearby fields more frequently than English speakers, who discussed dairies and manure fertilizer more frequently. For example, one Spanish speaker was more worried about pesticide use near her property than cattle:

*“Where I live there is a property that only had some cows, but the neighbor rented it out to them, for the hops, and now I feel that they are going to use pesticides. Maybe they did not use them before, so I had no pesticide danger, but now I do.”*

### Home repair experience

Spanish-speaking participants shared their experiences investigating well water issues and asked questions about how to make repairs on their own. Several Spanish speakers raised concerns about discolored or foul-smelling water and deposits left on faucets, pipes, and appliances, as did several English speakers. Several Spanish speakers identified aging piping components as a potential source of contamination. Concerned about the look and smell of their water, these participants described the actions they had taken to address these issues: opening pipes, discovering extensive corrosion, consulting neighbors, flushing water lines with chlorine, and researching anti-corrosion pipes online. Additionally, one Spanish speaker shared detailed observations of his employer’s well renovation project:

*“I saw when they took out the steel pipes [ …][and] put in another type of material. Since then I was thinking, because if we [live] about half a mile from where he has his well and I could see the tubes that are about this thick, they are like 7 tubes deep and each is about 15 or 20 feet long. [ …]we can see that although they are made of steel they are falling apart, they are very rusty. That’s why I get the idea that it’s necessary to do that.”*

Spanish-speaking participants also discussed septic tanks as a potential source of contamination in their wells, reflecting on the proximity of their well to neighboring septic tanks and the need for regular maintenance.

### Lack of actionable information

Throughout each focus group, participants raised questions about how to prevent and mitigate well water contamination. For example, one female participant in an English language group described her need for general well stewardship education:

*“My father, he is the one that did the maintenance on [the well], put whatever he had to do to make sure that the water was good. He passed away two years ago on [DATE]. Now, I’m new. I saw what he, you know, certain things that he used to do. I don’t know anything else, so how do I know if it’s good?”*

Spanish-speaking participants sought information to address the deposits, discoloration, and foul smells they observed in their well water. Spanish speakers asked questions about how to replace corroded pipes/well components and seek financial support for well renovations. Some asked questions about basic well water treatment, describing situations that could be resolved with particle filters and water softeners. Others expressed confusion about the purpose of different treatment systems.

Some participants shared that their wells had been tested once or twice in the past. Some learned that they had elevated nitrate levels or bacterial contamination. Others learned that their water was safe to drink, and among this group some participants still sought information about future contamination or contaminants that had not been tested. Before reading the fact sheet on testing, participants were asked specifics about the testing procedure, including recommended contaminants and testing frequency. Most participants across the four focus groups were unaware of government recommendations to test every year for nitrate and total coliform. One participant reported testing his well water for nitrate every year, but stated that he did not test for total coliform. Many participants did not know testing costs or who to contact for testing. Even participants who had tested their wells in the past admitted that they had little knowledge of these specifics. Many had their wells tested by government agencies during groundwater monitoring studies, and so had little knowledge of how to test on their own. One female participant in a Spanish language group explained this when the moderator asked if participants knew how to test their well water:

*“No. We do not know, and like when the lady, one day they were doing it for free. But it is to just to know and they said [the water] was fine, but no, we do not know how to do it.”*

Lastly, participants who had not tested previously were unfamiliar with the testing process itself, asking if they could purchase a home testing kit or if an inspector would conduct the test at their house. Many participants discussed the need for more education in their communities or expressed gratitude for the information they had received during the focus group sessions.

### Desire to protect family

Many participants expressed worry or fear of sickness from contaminated well water and discussed the need to protect children, pregnant women, and older adults from contaminated well water. Many participants described testing, buying filters, being aware of contamination, and renovating wells as ways to protect their family’s health. Participants across all four focus groups expressed a desire to protect their families, but participants in one Spanish language group invoked personal responsibility. When discussing the need for information on well water contamination, a female participant in this focus group said,

*“The responsibility always ends with us. We are the owners of our family, of our children, and we are the ones who have to look for what we should do.”*

Although participants correctly identified vulnerable family members, they rarely discussed specific health effects unprompted. This worry about sickness seemed to be based on what could happen rather than knowledge of specific water-related health effects. An older male participant in an English language group shared,

*“I tried to monitor my health yet not be paranoid. I just went through a nasty gallbladder operation. Anything that happens to me, I wonder is it just getting old or is there something hurting me? [ …]I’m not sixteen anymore.”*

In contrast, several participants stated their well water had positive or neutral effects on their health. Several said that when a family member gets sick, they drink more well water.

### Economic limitations

After reading the fact sheet and learning about well water testing, participants were asked what makes it difficult to test well water. The most common response from participants across all focus groups was financial cost. Participants in three focus groups stated that taking time off work was also a barrier to testing. One female Spanish speaker described how substantial socioeconomic challenges take priority over water quality for community members who have immigrated from Mexico.

*“When you arrive here all you do is think: ‘Tomorrow I have to work, I have so much to do to eat,’ [ …]but [the water] is the least you think about sometimes.”*

Additionally, several English speakers shared that installing and maintaining water treatment systems was expensive. One individual recalled a recent program in the area, sharing that even when a reverse osmosis system is installed for free, the recurring costs of filter replacements are too burdensome for local families. This participant described how his family tended to purchase bottled water because it was cheaper than replacing the filters in his reverse osmosis system. Many participants stated that they drank bottled water, including several that reported using reverse osmosis systems or filters. No participants compared the value of testing and treatment to that of bottled water, but several Spanish speakers shared that bottled water is expensive. Additionally, two English-speaking participants expressed that the costs of yearly testing and reverse osmosis installation were worthwhile in order to protect their families.

### Lack of technical support

Participants across all focus groups shared their difficulties with the technical aspects of well stewardship, including interpreting water quality test results, selecting appropriate mitigation actions, and maintaining POU/POE treatment systems. Participants who had tested their wells stated that their water quality test results were difficult to understand, sharing that they did not know the water quality standards and whether those standards were protective of infants and children. After reading the fact sheet on nitrate and coliform bacterial testing, several participants asked how they could use contaminated water (e.g. cooking, bathing) and how to improve their water quality. Participants expressed that testing laboratories did not explain how to read water quality reports, and provided insufficient guidance on how to address contaminated well water. Several English speakers described difficulties navigating services after testing their well. Speaking about the process to disinfect a well with bacterial contamination, one female English-speaking participant stated,

*“So I did some digging and the process seemed very scary so I didn’t even try to [do it] [ …]but I don’t know who to pay to come and do it.”*

Across all focus groups, participants stated that they had some type of POU or POE treatment system, but still did not drink the treated well water. Several expressed suspicion or confusion about whether the treated water was safe to drink. Greatly frustrated, one English-speaking participant described her difficulties treating *E. coli* in her well, “playing phone tag” with treatment companies, implementing various treatment methods, and still not being satisfied with the quality of her well water. “I still don’t like it,” she said and concluded, “I [am] literally thinking of selling my house and getting out of here.” Several participants also mentioned other challenges with their treatment systems, including the inconvenience of replacing filters, reduced water pressure, and changes in taste.

### Landlords and neighbors

Finally, participants described how landlords and neighbors can make it difficult to secure safe well water. At the start of one Spanish language group, a renter asked with great concern whether she or her landlord was responsible for ensuring the safety of her water. Another participant, one who knew the renter outside of the study, commented that the renter’s water smells terrible. The renter explained that her landlord “doesn’t want to help.” The renter was grateful to hear that she could test her well water quality independent of her landlord. An English-speaking participant who shared her well with her neighbor asked whether her neighbor’s cattle, which he kept near the well, could contaminate the well water. She also described barriers to treating her well for bacterial contamination, explaining that she did not treat the well because her neighbor drank bottled water and was not interested in treatment. Recalling her thought process at the time, she said, “Well, I’m not doing anything if the neighbor’s not doing it.”

## Discussion

The purpose of this study was to identify the factors that impact private well water testing behavior within predominantly Latino communities in rural, agricultural areas. Emergent themes extended beyond the topic of testing and included other well stewardship behaviors, such as well maintenance and treatment. Participants in this predominantly Latino community reported factors that are commonly observed in other rural communities, corroborating existing literature on well stewardship [11–16]. Additionally, two themes emerged that have not been reported in well stewardship literature: landlords and neighbors and home repair experience. Although many of these factors have been reported previously, our study findings suggest that the cumulative burden of these factors may make well stewardship unsustainable in rural, agricultural Latino communities.

Lack of actionable information arose as a factor that may hinder well stewardship. Many participants were concerned about well water contamination, but they lacked the actionable information needed to test and treat their well water. Literature indicates that some perceived risk factors, such as the belief that one’s household is at risk for drinking contaminated water, are not significant predictors of well testing behavior, but action knowledge, such as knowing who to contact for testing, is [14]. Additionally, participants discussed the need for more well stewardship education in their communities. Based on these findings, we recommend that state and local health agencies make actionable information on well water testing, maintenance, and treatment easier to access for both Spanish and English-speaking communities.

Home repair experience emerged as a factor that may support well stewardship, and has not been reported in the literature previously. Spanish-speaking participants described how they investigated water quality issues in their homes and asked detailed repair questions, suggesting that they have home repair experience. These individuals may be quick adopters of well maintenance behaviors because they appear to have the foundational skills needed to maintain their homes. In combination with well maintenance knowledge, these home repair skills may help build self-efficacy, which is a significant predictor of well maintenance behaviors such as visual well inspection [18]. Educational resources geared towards well users with home repair experience may improve rates of well stewardship behaviors in communities like this one. Home repair experience did not emerge as a theme in the English language groups, and further research is needed to determine how home repair experience may differ across language groups.

Economic limitations and lack of technical support emerged as factors that may hinder well stewardship. For example, participants reported that POU/POE treatment systems were burdensome because they were costly and inconvenient, and participants were not confident that treated water was safe to drink. Reverse osmosis systems are one of the POU/POE systems recommended by the CDC for long-term nitrate removal and are highly efficacious [19]. However, POU/POE systems are only effective solutions if the appropriate system is selected for the contaminant of interest [15], and they are maintained properly over time [27]. In low-resource areas, challenges around POU/POE systems are amplified when combined with existing economic burdens. Globally, POU/POE systems are not scalable solutions for water quality issues at current prices and user preferences [28]. Domestically, POU/POE systems have limited adoptability in rural Latino communities along the US-Mexico border, commonly called *colonias* [29]. In these low-income communities, only 25% percent of households surveyed were willing to adopt POU/POE systems if they cost $10 to $100 [29]. Asking residents under economic stress to maintain this technology without financial assistance and sustained technical support may not be a sustainable solution in rural Latino communities.

The majority of participants purchased bottled water, including several that also reported also using a reverse osmosis or other POU/POE system. Although bottled water is recommended by the CDC as a short-term solution to well water contamination [19], the drawbacks of long-term bottled water use are many: bottled water is more expensive than piped water; its quality is not well regulated; it may not be used consistently enough to prevent disease; and the resulting waste has significant environmental impacts [30]. Similar to LYV communities, rural Latino communities along the US-Mexico do not have access to piped municipal water and rely heavily on bottled water for drinking and cooking [29, 31]. Households in these communities were classified as water insecure, a measure that assesses how water reliability, quality, and affordability impact basic human activities [32]. Water insecurity may be a useful metric for assessing the impact of bottled water use and well water contamination on everyday life within Latino communities in agricultural regions as well.

This study observed that landlords and neighbors may be important factors impacting well stewardship but have received little attention in the well stewardship literature. The landlord-related challenges reported by study participants may help explain the observed association between rentership and lower testing rates [17]. Renters face similar challenges with their landlords when addressing asthma triggers in their homes, including a lack of knowledge about tenant rights, lack of financial resources, and a fear of retribution [33]. Policy interventions may be necessary to address landlord-related barriers to well stewardship. The New Jersey Private Well Testing Act requires landlords to test private well water quality once every 5 years and notify tenants of the test results [34]. Washington and other states could benefit from similar legislation, particularly if required testing frequencies were aligned with CDC recommendations, such as annual testing for nitrate [19]. Additionally, Krieger et al. observed that landlords are more responsive to renters’ requests when public health staff encourage them to correct hazardous housing conditions [35]. Negotiation assistance for well water issues with landlords and shared well users could be incorporated into the duties of public health nurses, environmental health professionals, and community health workers.

The RANAS factor blocks that were dominant in the focus group results were risk, attitude, and ability. Participants’ concerns about water quality and their knowledge of agricultural sources of contamination appeared to align with the risk block. Participants’ desires to protect family members and their perceptions of the financial costs of well stewardship appeared to align with the attitude block. Participants’ home repair experience appeared to align with the ability block. Economic limitations, lack of technical support, and landlords and neighbors also seemed to limit participants’ abilities to complete well stewardship behaviors. However, these themes did not appear to align clearly with the ability factors in the RANAS model, i.e. action knowledge, self-efficacy, maintenance self-efficacy, and recovery self-efficacy. These three themes seemed to extend beyond the individual well user and describe well stewardship factors that operate at the community or societal levels. It is logical that the RANAS model, an individual-level framework for behavior change, would not capture these themes. Additional research is needed to further characterize community and societal-level factors of well stewardship, and determine their impact relative to individual-level factors. Researchers may benefit from Balazs and Ray’s Drinking Water Disparities Framework, a multilevel framework that expands the traditional exposure-disease model for environmental health outcomes to include actors at the household, community, and regional levels [36].

Traditional well stewardship interventions, which typically promote individual behavior change, often increase socioeconomic disparities [37] and may have limited impact in rural, agricultural communities like the LYV. Research in rural Latino communities in California’s Central Valley suggests that interventions implemented at the community, county, or state level may reduce water contamination exposure more effectively and equitably than household-level interventions [36]. There is significant evidence that piped municipal water is the best method for reducing poverty and improving public health [38, 39], and until such infrastructure is developed, policies are needed to reduce well stewardship burden for communities like the LYV. Universal water testing, by way of regulatory requirements and income-based subsidies, may be an important step towards reducing disparities in well water contamination exposure [40]. Additional programs would be needed to support residents with contaminated wells, and could include subsidies for POU/POE installation/maintenance or well replacement alongside sustained technical support.

Although focus group questions centered around well water testing, participant discussions often shifted to well water treatment and prevention of contamination. These observations suggest that private well users prioritize solutions to contamination issues when seeking information on well stewardship. Additionally, although the study question focused on nitrate and its connection to agricultural communities, focus group themes were not nitrate-specific. Nitrate-specific research is needed to explore the connection between agriculture, water quality, and rural Latino communities, but research on well stewardship interventions should be inclusive of all contaminants that pose a health risk to local communities.

This study had several limitations and strengths. The perspectives shared by focus group participants may not be representative of rural, agricultural Latino communities due to purposive sampling methods and inclusion of non-Latino participants. Although we feel we reached saturation on the universal themes across all focus groups, we did not reach saturation on the differences between language groups. Additionally, in this exploratory study, a minority of participants identified as renters or shared well users. The role of language, landlords, and shared wells should be explored in future research. To our knowledge, this was the first study to explore private well stewardship in a rural, agricultural Latino community. Another strength of this study is a strong collaboration with a bilingual and bicultural team member from the community whose knowledge of Latino culture and historical groundwater issues in the region increases the validity of the study results.

## Conclusion

Private well users in this rural, agricultural community with a predominantly Latino population may have many of the same factors that impact well stewardship behavior in other communities. However, the cumulative burden of well stewardship in rural Latino communities may make well stewardship unsustainable. Community, county, or state interventions may be needed to equitably prevent exposure to well water contaminants. Additionally, this study observed home repair experience and landlords and neighbors as factors that may impact well stewardship behavior. These factors have not been reported in the literature previously and represent opportunities for future research. This study can inform well stewardship policies and programs in this and other rural, agricultural Latino communities.

## Supplementary information

**Additional File 1.** Agency fact sheet on private well water read with focus group participants. The file lists the agency that developed the fact sheet, modifications to the sheet for study purposes, and which portions of the sheet were read with focus group participants.

## Data Availability

Data files and materials pertaining to this publication are available from the corresponding author upon reasonable request.

## Abbreviations

CDC: Centers for Disease Control and Prevention
NIOSH: National Institute for Occupational Safety and Health
NIH: National Institutes of Health
NWCPHP: Northwest Center for Public Health Practice
LYV: Lower Yakima Valley
POE: point-of-entry
POU: point-of-use
SD: standard deviation
USGS: United States Geological Survey
