# Supplementary material for: Private well stewardship within a rural, agricultural Latino community: a qualitative study
Source: BMC Public Health. 2020 Jun 5;20:863. doi: 10.1186/s12889-020-08963-4 (PMC7275588; doi:10.1186/s12889-020-08963-4)
Supplement: Supplementary file 1 — Additional File 1. Agency fact sheet on private well water read with focus group participants. The file lists the agency that developed the fact sheet, modifications to the sheet for study purposes, and which portions of the sheet were read with focus group participants. [file 12889_2020_8963_MOESM1_ESM.pdf]

Additional File 1: Agency fact sheet on private well water read with focus group participants

The fact sheet read by focus group participants was the Washington State Department of Health document number 333-171. The agency's logo was removed and the contact information for the local health district was added to the fact sheet. The red boxes indicate which portions of the fact sheet were read with focus group participants.

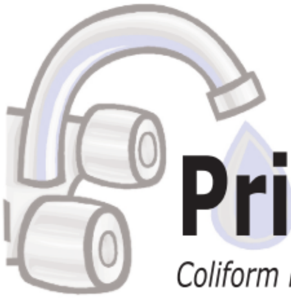

Private Well Water

Coliform Bacteria and Nitrate Information for Private Well Users

Why should my well water be tested?

Drinking contaminated water is a health risk. Some contaminants cannot be seen, smelled, or tasted. Two of the most common contaminants in drinking water are coliform bacteria and nitrate and they can be harmful.

Who should be testing my well water?

You or your landlord. Private well users are responsible for testing their own water. If you don't own your home but you use a private well, talk with your landlord about getting your water tested or seeing the most recent results. You can always take a water sample yourself and have it tested.

What should I test for and how often?

The Department of Health recommends that you test your private well water every year for coliform bacteria and nitrate.

You should also test your water when:

- You notice a change in your water, such as taste, color, or smell.*
- Your well has been flooded.
- You replace any part of your well system.
- Someone in your household is pregnant, nursing, or has an unexplained illness and you suspect your water may be at risk.
- You hear that a neighbor's water is contaminated.
- You live near industrial or agricultural activities.*

*These may require testing for something other than coliform or nitrate.

If you have had previous contamination problems or are concerned about specific contaminants, you may want to test your well water more often.

Where do I go to get my water tested?

Certified drinking water labs are located across the state. The lab you select or your local health department can help you decide what to test for, how to collect samples, and how to understand results. There is a cost for these tests. Costs this year (2018) range from $20 to $28 per test for coliform bacteria, and $18 to $48 per test for nitrate. Most labs like to provide their own sample bottles.

My nitrate level is *less than* 10 ppm, what should I do?

Nitrate levels can vary throughout the year, so if your level is 5 ppm or higher, you may want to re-sample in six months.

My nitrate level is *more than* 10 ppm, what should I do?

If your nitrate test shows levels higher than 10 parts per million, find a different and safe drinking water supply. The quickest thing to do is to begin using bottled water for drinking and food preparation. Do NOT boil water with high nitrate. Boiling water may actually increase the nitrate level, making the problem worse!

Another option is to install a device or filter designed to remove nitrate from your water. These devices are often installed on kitchen faucets, where people get their water for drinking and cooking. Nitrate is not absorbed through the skin, so it is safe to clean and bathe with it.

Other, longer term solutions include:

- Drilling a deeper well into a different groundwater source;
- Connecting to a public water system; or
- Working with others in your community to develop a new public water system to serve your home and nearby neighbors.

My test results came back with coliform in the water, what should I do?

Coliform tests usually come back as SATISFACTORY or UNSATISFACTORY. If you receive a SATISFACTORY report, it means your water was free of these bacteria at the time of the sample. Be sure to test every year for coliform bacteria.

If you receive an UNSATISFACTORY report, it may be contaminated. Do not drink the water until it tests SATISFACTORY. Find a different and safe drinking water supply. The quickest thing to do is either begin using bottled water or boil all water for drinking and food preparation. This also includes water used for making ice or coffee, brushing teeth, and washing fruits and vegetables you eat raw. Boiling water rapidly for one minute usually kills bacteria.

Your lab and local health department can help you determine if you should resample, disinfect your well, or take other action based on your results.

What are coliform bacteria and why should I care?

Coliform bacteria are organisms that are present in the environment and in the feces of humans and animals. Coliform bacteria will not likely cause illness, but their presence in drinking water indicates disease-causing organisms may also be present.

What is nitrate?

Nitrogen is a chemical found in most fertilizers, animal manure, and in septic tanks. Natural bacteria in the soil can change nitrogen into nitrate. Rain water and irrigation water can carry nitrate down through the soil into the groundwater.

What can nitrate do to me?

Too much nitrate in your body makes it harder for red blood cells to carry oxygen. While many people do not notice a difference, this can be very dangerous for infants and pregnant women. Infants exposed to high amounts of nitrate may develop "blue –baby syndrome," a condition that is rare but can be fatal.

What are the symptoms of blue-baby syndrome?

Symptoms can be confused with other illnesses. An infant with mild to moderate blue-baby syndrome may have diarrhea, vomiting, and be lethargic.

In more serious cases, the infant may have:

- skin that becomes gray, darker brown, or blue, or
- lips, finger or toe nails with a blue-like color, or
- trouble breathing.

My test results came back with *both* coliform and nitrate, what should I do?

Find a different and safe drinking water supply. The quickest thing to do is to begin using bottled water for drinking and food preparation. Boiling water kills coliform bacteria, but does not remove nitrate. Do NOT boil water with both coliform and nitrate. It may increase the nitrate level, making the problem worse! See other options under nitrate and coliform above.

My test results came back OK, but I don't like the taste/smell/ appearance of my water. What is wrong with it?

Some contaminants make water smell, taste, or look bad but are not harmful to your health. Your lab and local health department can help you determine if you need to test or treat your water.

What about Home Water Treatment Units? I've heard that these can help.

Point of use (POU) filter systems treat water at a single tap. Point of entry (POE) filter systems treat water used throughout the house.

Three types of systems that can remove nitrate from your water are:

- Reverse Osmosis Unit
- Distillation Unit
- Anion Exchange Unit

Important: All POU and POE filter systems or treatment units need maintenance to operate effectively. If they are not maintained properly, contaminants may accumulate in the units and make your water worse. In addition, some vendors may make claims about their effectiveness that are not based on science. The EPA does not test or certify treatment units, but two organizations that do are NSF International and Underwriters Laboratory.

How can I protect my well water from contamination?

Make sure your wellhead extends 6 to 12 inches above the surface of the ground and is capped to keep contaminants out. Seal the ground around the wellhead and slope it away so water does not collect and seep into the well.

It is important to keep your well safe from potential contaminants that may be around your home. The further away from contamination sources, the better.

Experts suggest your well should be at least:

- 50 feet from a septic tank,
- 100 feet from the edge of a drainfield, fuel tank, barn, and any storage shed for fertilizers and pesticides, and
- 250 feet from a manure stack.

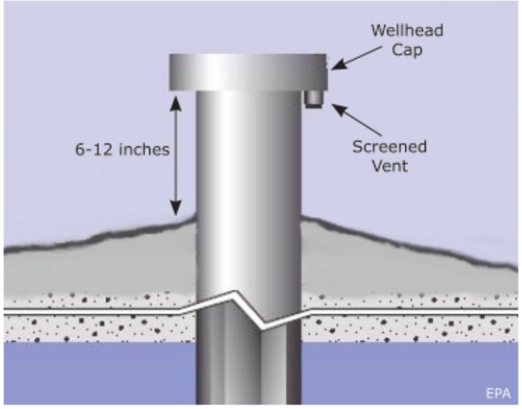

Potential Well Contaminants

1. Septic Tank
2. Household Wastes
3. Livestock Wastes
4. Pesticides and Fertilizers
5. Landfills
6. Local Industries
7. Underground Storage Tanks

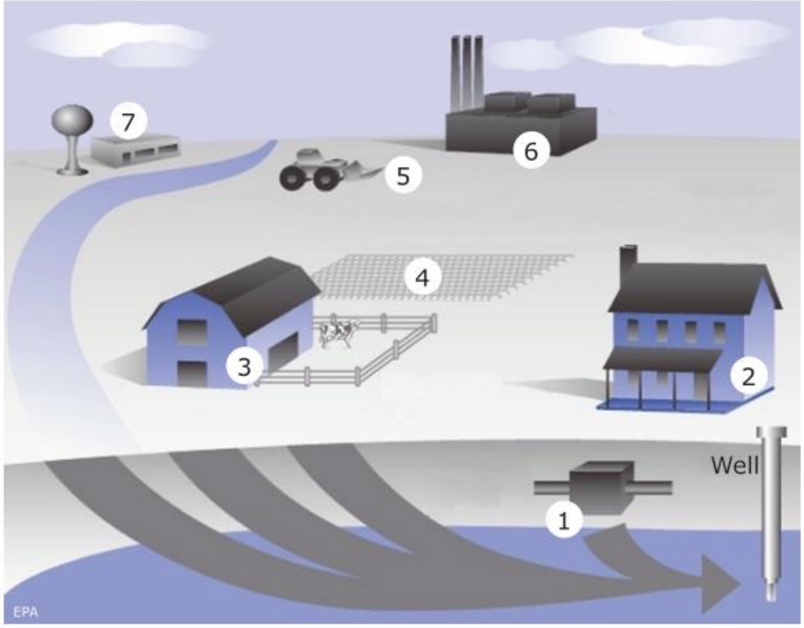

Questions about your well?

Yakima Health District
Help Desk Line
509-249-6508

Additional Resources

Local Health Departments
www.doh.wa.gov/LHJMap/LHJMap.htm

Certified Labs in Your Area
www.ecy.wa.gov/apps/eap/acclabs/labquery.asp

Certifying Organizations for Home Water Treatment Units
NSF International (Formerly National Sanitation Foundation), www.nsf.org
Underwriters Laboratory, www.ul.com

Center for Disease Control and Prevention Publications
Private Wells, www.cdc.gov/healthywater/drinking/private/wells/location.html
Emergency disinfection of wells, http://emergency.cdc.gov/disasters/wellsdisinfect.asp

Environmental Protection Agency Publications
Household wells, www.epa.gov/safewater/privatewells/pdfs/household_wells.pdf
Secondary Standards, www.epa.gov/safewater/consumer/2ndstandards.html
Filtration Facts booklet, www.epa.gov/safewater/faq/pdfs/fs_healthseries_filtration.pdf
Source Water Protection, http://cfpub.epa.gov/safewater/sourcewater

For persons with disabilities, this document is available in other formats.
To make a request, call 1-800-525-0127 or 1-800-833-6388 (TTY/TDD).
